# Altered effective connectivity of resting state networks by acupuncture stimulation in stroke patients with left hemiplegia: A multivariate granger analysis

**DOI:** 10.1097/MD.0000000000008897

**Published:** 2017-11-27

**Authors:** Cai-Hong Fu, Kuang-Shi Li, Yan-Zhe Ning, Zhong-Jian Tan, Yong Zhang, Hong-Wei Liu, Xiao Han, Yi-Huai Zou

**Affiliations:** a Department of Neurology and Stroke Center, Dongzhimen Hospital, the First Affiliated Hospital of Beijing University of Chinese Medicine, Beijing, China; b Shunyi Hospital Affiliated to Beijing Hospital of Traditional Chinese Medicine; c Department of Emergency, Beijing GuLou Hospital of Traditional Chinese Medicine; d The National Clinical Research Center for Mental Disorders & Beijing Key Laboratory of Mental Disorders, Beijing Anding Hospital, Capital Medical University; e Department of Radiology, Dongzhimen Hospital, the First Affiliated Hospital of Beijing University of Chinese Medicine, Beijing, China.

**Keywords:** effective connectivity, granger analysis, hemiplegia, resting state networks, stroke

## Abstract

The aim of this study was to explore the response feature of resting-state networks (RSNs) of stroke patients with left hemiplegia by acupuncture stimulation.

Nineteen stroke patients with left hemiplegia and 17 controls were recruited in this study. Resting-state functional magnetic resonance imaging data before and after acupuncture were acquired using magnetic scanning. The independent component analysis (ICA) was employed to extract RSNs related to motion, sensation, cognition, and execution, including sensorimotor network (SMN), left and right frontoparietal network (LFPN and RFPN), anterior and posterior default mode network (aDMN, pDMN), visual network (VN), and salience network (SN). Granger causality method was used to explore how acupuncture stimulation affects the causality between intrinsic RSNs in stroke patients. Compared with healthy subjects, stroke patients presented the more complex effective connectivity. Before acupuncture stimulation, LFPN inputted most information from other networks while DMN outputted most information to other networks; however, the above results were reversal by acupuncture. In addition, we found aDMN reside in between SMN and LFPN after acupuncture.

The finding suggested that acupuncture probably integrated the effective connectivity internetwork by modulating multiple networks and transferring information between LFPN and SMN by DMN as the relay station.

## Abstract 2

Supplemental Digital Content is available in the text

## 1 Introduction

Motor disorder is the most prominent symptom in ischemic stroke, severely affecting patients’ quality of life, as well as aggravating global health burden in stroke rehabilitation and nursing.^[1,2]^ Therefore, promoting motor function recovery of the hemiplegic limb and improving living quality of patients is an urgent question in the field. Brain plasticity has been proven by various studies including clinical trials and animal studies.^[3–5]^ Some researchers have reported that impaired motor function in patients with acute cerebral apoplexy may exhibit spontaneous improvement to some extent, which is often considered as the result of brain functional reorganization.^[6,7]^ The research on mechanism of brain plasticity following stroke has become a hot topic at home and abroad.

In recent years, numerous evidences indicate that the restoration of motor function after stroke is associated with brain plasticity. It refers to functional compensation of the contralateral brain, functional reorganization of the adjacent, and remote regions with regard to the ischemic lesion.^[6,8]^ Studies on functional magnetic resonance imaging (fMRI) have found that brain plasticity could be manifested in changes of cortical thickness, gray matter volume, white matter fiber tracts, brain activation, and functional connectivity (FC).^[9–12]^ A growing number of studies have revealed that a human brain still exists spontaneous and rhythmic neuron activity in resting state, which demands a bigger share of energy consumption than accepting an external stimulation or performing a task.^[13–15]^ Meanwhile, resting-state fMRI (rs-fMRI) is particularly suitable for stroke patients usually accompanied by cognitive impairment or motor disabilities without specific tasks.^[16]^ Indeed, rs-fMRI studies have become the main trend of stroke research in recent years. So far the characteristic of brain regional properties or inter-regional FC of the ischemic stroke patients have been widely concerned,^[7,17,18]^ however, a relatively small number of studies focus on the resting-state brain network.

Raichle et al found some active brain functional networks independent and inter-related in resting state, which cooperate to maintain the brain activity.^[14]^ In fact, the clinical manifestation of patients with cerebral infarction is complicated, it has been seen not just in dysfunction of motor but in a series of symptoms, such as visual, auditory, spatial attention, and cognition. The above-mentioned discoveries indicate that focal cerebral infarctions may lead to abnormal changes of multiple brain networks at rest. Thus, the concept of brain networks restructuring in stroke patients, which mainly refers to directly causing damage to fiber pathway by infarction lesion, further influence the integrity of network structure among the bilateral cerebral hemisphere.^[19,20]^ To strengthen the study on the mechanism of stroke hemiplegia from the perspective of resting-state networks (RSNs) not only can reveal the mechanism of brain networks remodeling, but also have great constructive significance for the selection of rehabilitation methods.

Present research work focuses on the characteristic of FC of these networks including sensorimotor network (SMN), auditory network (AN), executive network, and default mode network (DMN) associated with movement, perception, and cognitive function.^[21–23]^ The connectivity of SMN area showed asymmetrical changes during motor recovery in stroke patients. Tuladhar and his colleagues^[23]^ also found reduced FC within DMN in patients following “first ever” stroke compared with health controls. Yet, it is unclear how these brain networks interact each other in patients with apoplectic hemiplegia. Granger causality (GC) is an important method of effective connectivity (EC) analysis. In contrast to FC, EC can reflect the directional influence of one brain region (or network) on another, which has been gradually applied to various kinds of neural diseases or psychiatric disorders, such as epilepsy, multiple sclerosis, and schizophrenia.^[24–26]^ The latest studies have demonstrated attenuated EC between cortical and subcortical areas in stroke patients during passive motor task.^[27]^ However, the EC between resting-sate brain networks has been little studied.

It is generally known that acupuncture therapy can promote stroke rehabilitation,^[28]^ but its mechanisms underlying brain networks plasticity remain unclear. In this study, the Granger causality method (GCM) was used to test our hypothesis that acupuncture may regulate multiple resting-state brain networks in stroke patients with hemiplegia. Then we sought to investigate the mechanism of acupuncture therapy in promoting brain network reorganization with Yanglingquan (GB34) commonly used in hemiplegia rehabilitation as cut-in point.

## 2 Materials and methods

### 2.1 Subjects

**Figure 1:**
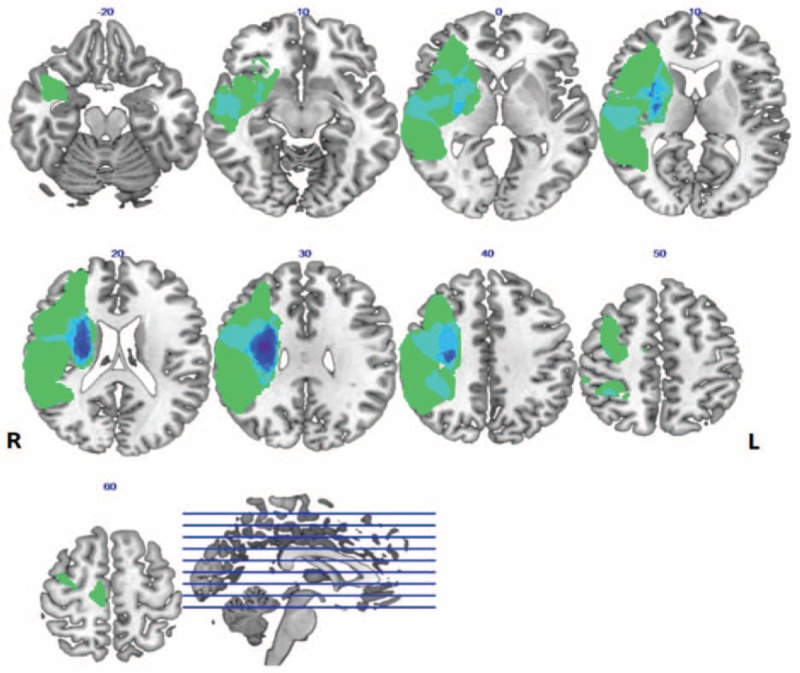
Distribution of the lesion areas for all stroke patients with hemiplegia. The lesion area overlap across stroke patients was rendered on the brain. Colors represent the number of patients with a lesion to a specific voxel. Numbers above each axial map and sagittal map represented the coordinate values of the lesion areas corresponding brain layers. Letters L and R correspond to the left and right sides of the brain, respectively.

A total of 19 right-handed patients with ischemic stroke (12 males; mean age: 61.53 ± 8.92 years) were enrolled from Dongzhimen Hospital. The demographic and clinical information are summarized in Table 1, and the overlap lesions of all stroke patients are exhibited in Figure 1. The inclusion criteria were as follows: first onset of ischemic stroke; being left hemiplegia; the lesion restricted to the internal capsule and neighboring regions in the right hemisphere; age range from 35 to 75 years old; 2 weeks to 6 months after the onset of stroke; being unconscious retardation and stable condition. The exclusion criteria were as follows: recurrent stroke; lesion involved in bihemispheric or brain stem infarcts; being pregnant or lactation; any other brain structure damage identified by MRI examinations; any history of alcohol or drug dependency; any other health problems or poor physical conditions that may influence participation; any MRI contraindications. Another 17 healthy subjects (9 males; mean age: 53.94 ± 4.83 years) were recruited for this study as healthy controls. The study was approved by the Research Ethical Committee of Dongzhimen Hospital affiliated to Beijing University of Chinese Medicine and conducted in accordance with the Declaration of Helsinki. Written informed consents were obtained from all participants.

**Table 1:**
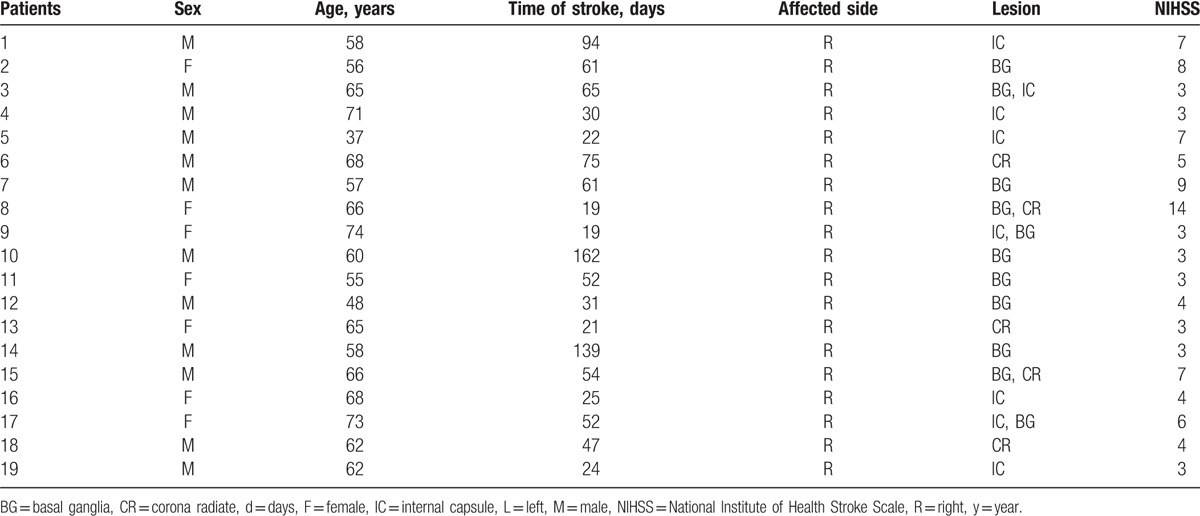
Demographic and clinical characteristics of stroke patients with hemiplegia.

### 2.2 Study design

Resting-sate fMRI (rs-fMRI) design paradigm was adopted in this study. In order to ensure the quality of brain imaging, participants rested on the bed for 5 minutes before scanning procedure and were informed to remain awake and calm, close their eyes, take slow breaths, keep the whole body stationary and relax, and avoid falling asleep or thinking. During the whole process, subjects’ heads were stabilized using foam pillows so as to prevent head movement and earplugs were applied to lower the noise produced by MRI scanner.

All stroke patients undergone 8-minute and 10-second resting-state scan preceded acupuncture stimulation. Then an experienced acupuncturists inserted the needles into the left GB34 of stroke patients and followed these procedures including 1-minute needle retention (only scanning without twirling), 1-minute needle twirling (only scanning), and 8-minute and 10-second resting-state scanning. The structural imaging sans were conducted on each participant for 4 minutes and 10 seconds. Notably, stroke patients carried out rs-fMRI scans before and after acupuncture intervention respectively while healthy controls had only once in this study.

### 2.3 Interventions

Based on the literature reports and clinical experience, Yanglingquan (GB34) was applied in the study as one of the most frequently used acupoints and proved to be effective for hemiplegia rehabilitation. Acupuncture was performed by vertically inserting a sterile, disposable, silver needle (40 mm in length and 0.40 mm in diameter) into the left Yanglingquan located in the sagged place anterior and inferior to the head of the fibula to the depth of 1.5–2.0 cm, with the certain frequency of twirling-rotating method (30 times per minute). In the whole process, acupuncture manipulation was performed by the same experienced acupuncturist.

After MRI scanning, the feeling of de-qi was evaluated for all subjects by the form of questionnaires including the feature (such as pain, numbness, and distension) and rating of the De-qi (the features mentioned above were divided into different types from 0 to 10 based on the degree of sensation, 0 stand for no feeling, 10 stand for unbearable).

### 2.4 MRI data acquisition

All MRI data were obtained using a 3.0 Tesla MRI scanner (Siemens, Sonata Germany) in the Radiology Department of Dongzhimen Hospital. Before scanning, participants were informed the objective and procedure of the experiment, as well as some of attentive matters in detail. The resting-state fMRI were acquired by adopting a T2-weighted singleshot, gradient-recalled echo planar imaging (EPI) sequence. The concrete parameters were as follows: TR = 2000 ms, TE = 30 ms, matrix = 128 × 128, FOV = 240 mm × 240 mm, slice thickness = 5 mm, number of layers = 26, and flip angle = 90°. Three-dimensional (3D) structural imaging of the whole brain was obtained using T1W1 sequence with acquisition parameters: TR = 1900 ms, TE = 3.93 ms, FOV = 240 mm × 240 mm, slice thickness = 1 mm, number of layers = 176 ± 5, and flip angle = 15°.

### 2.5 MRI data preprocessing

All MRI data were preprocessed using the Data Processing Assistant for Resting-State fMRI (DPARSF) software package designed by Beijing Normal University, Beijing, China.^[29]^ First, the original file format was transformed into NIFTI-1 data format (Neuroimaging Informatics Technology Initiative, National Institute of Mental Health, Bethesda, MD). In order to eliminate the influence of magnetic field inhomogeneity, data from 5 prior time points before and after acupuncture were removed from the resting-state images, respectively. Then slice timing and head movement were corrected for the residual phase sequence data. Functional imaging of each subject was performed based on the standardized diffeomorphic anatomical registration. The image data were aligned with the Montreal Neurological Institute template and resampled at 3 mm × 3 mm × 3 mm. Finally, the fMRI data were smoothed with 4-mm full-width-at-half maximum Gaussian kernel and detrended for avoiding time curve shifts caused by high temperature of an MRI scanner.

### 2.6 RSNs extraction using ICA

The method of group-independent component analysis (ICA) was adopted to identify and extract RSNs by using the fMRI Toolbox (GIFT) software (University of New Mexico, Albuquerque, NM).^[30]^ Based on all fMRI data (including stroke patients and healthy controls), the number of independent components in the package was estimated to be 20 by using the minimum description length (MDL) technique. To prevent sample order randomness from affecting ICA results, Randlnit and Bootstrap operations were repeated 20 times and the 20 independent components were individually calculated and evaluated using the *t* test. Then the principal component analysis and regression operation were further conducted to choose the relatively high model order ICA. Finally, based on the largest spatial correlation, the 7 independent components were inspected and selected visually with the resting-state brain network template widely reported in previous studies. In this research, we mainly analyzed and calculated the causal relations of these specific brain networks including left frontoparietal network (LFPN), right frontoparietal network (RFPN), anterior default mode network (aDMN), posterior default mode network (pDMN), SMN, visual network (VN), and salience network (SN).

### 2.7 Network causal relationship analysis

To investigate the causal relationships among the RSNs, functional network connectivity software^[31]^ was applied in this study. Seven RSN components identified by ICA were filtered at 0.01– 0.1 Hz and the generalized partial directed coherence was selected as the measured parameter. Then the multivariate Granger causal model (GCM)^[32]^ was utilized to explore the characteristics of EC among networks between the 0.01 and 0.1 Hz bandwidth. Briefly, the regression model of multivariate Granger causality analysis (GCA) was constructed based on time sequence components corresponding to the independent space components of brain networks as the input parameter of GCM. The order of the model was estimated by using group level optimization from the Akaike information criterion. A comparison between stroke patients and healthy controls, as well as stroke patients before and after acupuncture manipulation was analyzed on the causal interaction of 7 RSNs between any 2 networks. One-sample *t*-test for each group and 2-sample *t*-test for group comparisons were employed on all causal relationship among 7 networks. *P*-value was set as .05 for intergroup and intragroup comparisons. The results were displayed onto a 3D standard brain surface using BrainNet Viewer (Beijing Normal University).^[33]^

## 3 Results

### 3.1 Demographic and clinical information for stroke patients with hemiplegia

Nineteen right-handed patients with ischemic stroke (12 males; mean age: 61.53 ± 8.92 years) were screened in this study. The mean disease course was 53.05 ± 40.57 days and the mean NIHSS score was 5.21 ± 2.92. The details are summarized in Table 1 and the overlap lesions of all stroke patients are exhibited in Fig. 1.

### 3.2 The RSNs

**Figure 2:**
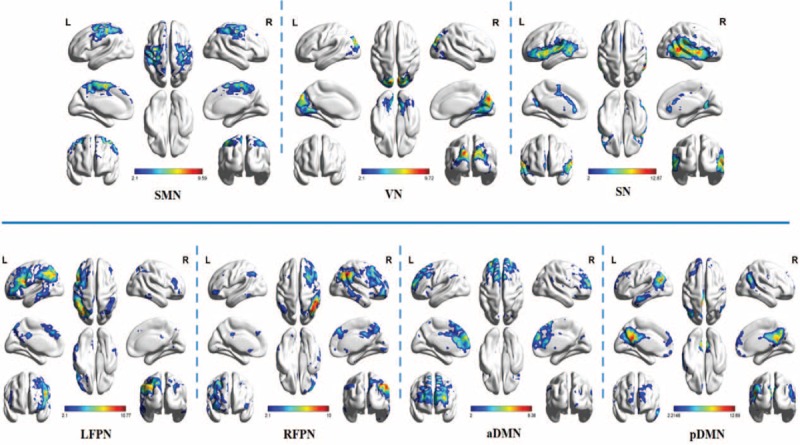
The RSNs extracted by ICA. The color scale represents the *t* values in each RSN. aDMN = anterior default mode network, ICA =  L = left, LFPN = left frontoparietal network, pDMN = posterior default mode network, R = right, RFPN = right frontoparietal network, RSNs = resting-state networks, SMN =  sensorimotor network, SN = salience network, VN = visual network.

Group ICA was applied to extract the resting-state brain networks based on all data of subjects (2 scan per patient before and after acupuncture manipulation, and 1 scan for healthy controls). Finally, 7 RSNs were identified and selected from 20 ICA components including 2 sensory processing networks (SMN, VN) and 5 cognitive networks (LFPN, RFPN, aDMN, pDMN, and SN) (see Fig. 2). The spatial distribution details of these networks are presented in the supplementary files.

### 3.3 Comparison between stroke patients and healthy controls in EC at resting state

**Figure 3:**
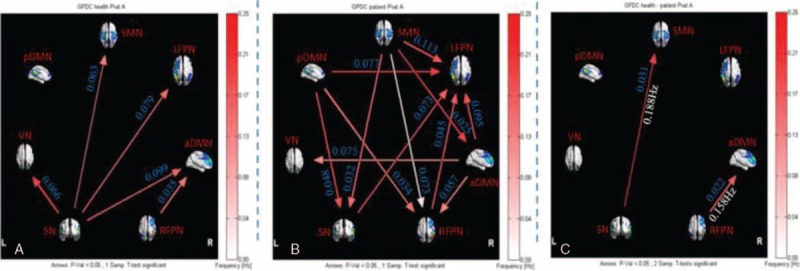
The resting-state effective connectivity comparison between stroke patients and healthy controls. (A) One-sample *t*-test result of intergroup intranetwork causal relationship of the healthy controls. (B) One-sample *t*-test result of intergroup intranetwork causal relationship of the stroke patients with hemiplegia. (C) Two-sample *t*-test result of intragroup intranetwork causal relationship of the healthy controls minus the stroke patients. Arrow directions represent causal effect between resting-state networks. Blue numbers indicate strength of causality. Right bands represent frequency domain, values on the color bar (corresponding with arrow colors) demonstrate frequency at which casual effect was found. White numbers represent frequencies of the causal connection. aDMN = anterior default mode network, L = left, LFPN = left frontoparietal network, pDMN = posterior default mode network, R = right, RFPN = right frontoparietal network, SMN =  sensorimotor network, SN = salience network, VN = visual network.

For healthy controls, 5 connections were found that had significant causal flow, including causal relationship between SN and LFPN, aDMN, SMN, VN, and between RFPN and aDMN. The results indicated that SN is an outflow hub at the junction of the resting-state brain networks. Unlike healthy subjects, stroke patients with hemiplegia presented more complex EC between brain networks. There were 12 connections with significant causal flow in stroke patients during resting state, as shown in Figure 3. Among these effective connections, RFPN, LFPN, and SMN constituted the center of the causal relationship. SMN outputted most causal information to other networks (LFPN, aDMN, RFPN, and SN) while bilateral FPN inputted most information from other networks (LFPN inputted information from SMN, pDMN, SN, RFPN, and aDMN, and RFPN outputted information from aDMN, SMN, and pDMN). In addition, the causal connections from pDMN to SN and from VN to aDMN were found. Compared with healthy subjects, there were significant differences in causal links between SN and SMN, and between RFPN and aDMN (*P* <.05). (Table 2 and Fig. 3)

**Table 2:**
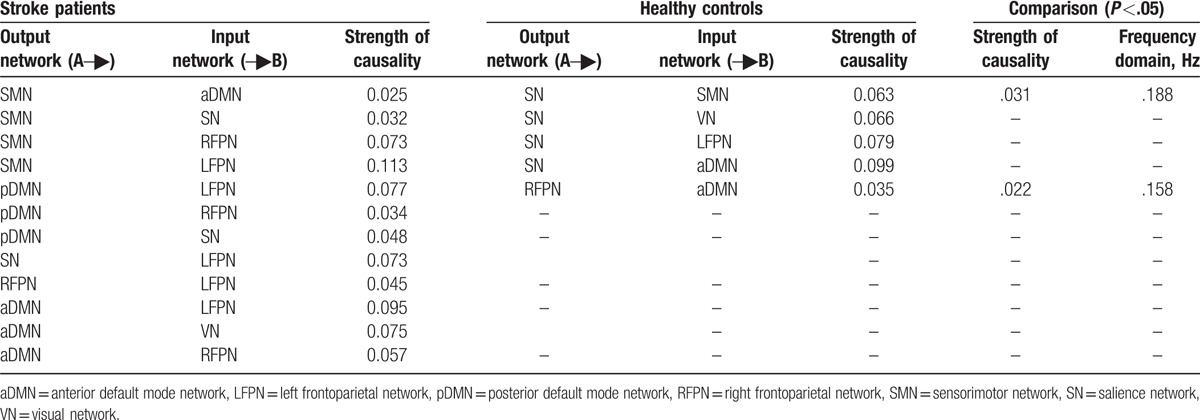
Altered effective connectivity among resting-state networks between stroke patients and healthy controls.

### 3.4 Comparison between preacupuncture and postacupuncture for stroke patients with hemiplegia in EC

**Figure 4:**
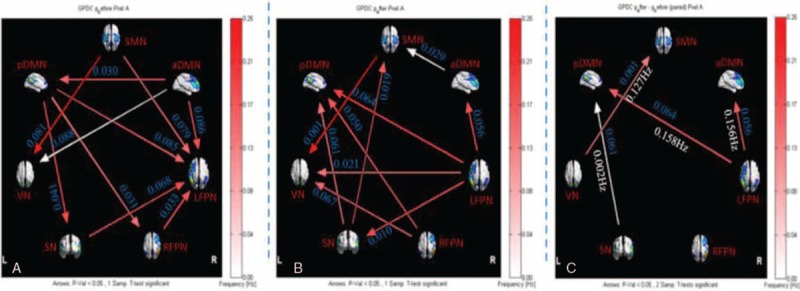
Intergroup and intragroup comparisons of the stroke patients before and after acupuncture. (A) One-sample *t-*test result of intergroup intranetwork causal relationship of the stroke patients before acupuncture. (B) One-sample *t*-test result of intergroup intranetwork causal relationship of the stroke patients after acupuncture. (C) Paired *t*-test result of intragroup intranetwork causal relationship of postacupuncture minus preacupuncture for stroke patients. Arrow directions represent causal effect between resting-state networks. Blue numbers indicate strength of causality. Right bands represent frequency domain, values on the color bar (corresponding with arrow colors) demonstrate frequency at which casual effect was found. White numbers represent frequencies of the causal connection. aDMN = anterior default mode network, L = left, LFPN = left frontoparietal network, pDMN = posterior default mode network, R = right, RFPN = right frontoparietal network, SMN =  sensorimotor network, SN = salience network, VN = visual network.

Before acupuncture intervention, stroke patients had 10 connections with significant causal flow. Among these causal connection between brain networks, LFPN inputted most information from other networks while DMN outputted most information to other networks before acupuncture; however, the above results were reversal by acupuncture. LFPN outputted most information while DMN inputted most information, details were as follows: acupuncture reversed the direction of EC between LFPN and aDMN, pDMN, SN, and turned over the direction of EC between pDMN and LFPN, RFPN, and SN. Meanwhile, these effective connectivities between LFPN and RFPN, SMN, between aDMN and pDMN, VN decreased while those between LFPN and VN, aDMN, between aDMN and SMN, between SMN and SN, between RFPN and VN increased. After acupuncture stimulation, LFPN outputted information to aDMN, then aDMN outputted the information to SMN, which formed the part of the information transmission loop. The finding suggested that acupuncture probably integrated the effective connectivity internetwork by modulating multiple networks. The result further confirmed the reversion of causality from aDMN to LFPN by acupuncture GB34 (Table 3 and Fig. 4).

**Table 3:**
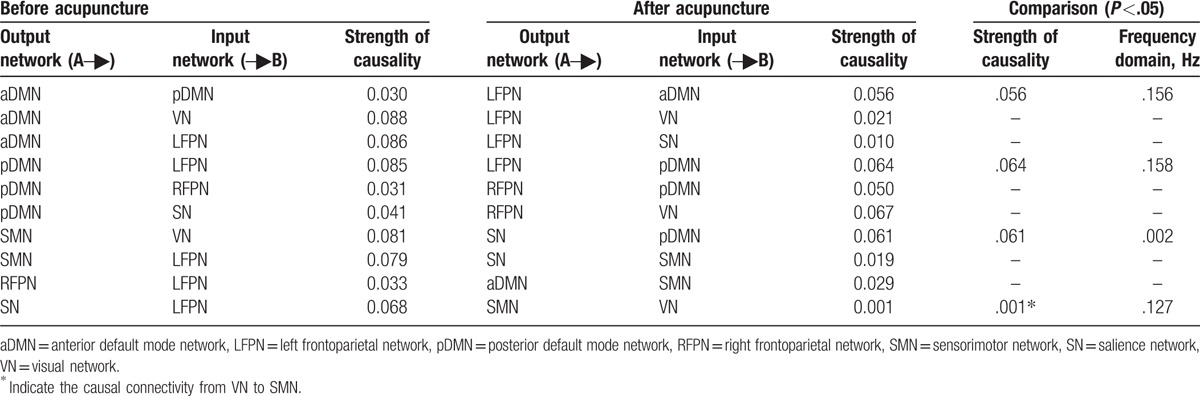
Altered effective connectivity among resting-state networks before and after acupuncture.

## 4 Discussion

To our knowledge, this study is the first report of brain plasticity mechanism of acupuncture in promoting hemiplegia from the perspective of multiple networks. We found that compared to healthy subjects, stroke patients with hemiplegia had more complex EC between brain networks, which mainly related to FPN, SMN, and DMN. Most importantly, these causal relationships between the RSNs were modulated and integrated by acupuncture at Yanglingquan, especially the transfer of information between advanced cognitive network and SMN.

Brain plasticity has been proved during stroke rehabilitation by several studies on animals and humans.^[3–5]^ Clinical practice indicated that stroke patients with hemiplegia not just displayed the damage of motor path, but manifested as a group of complicated syndrome including changes of cognitive, visual, and auditory function.^[34]^ Thus, the concept of brain networks reconfiguration in stroke patients has been put forward to interpret the possible mechanism of motor recovery related to improvement in cognitive function and spatial attention. Given this, we investigated the changes of EC between RSNs of stroke patients and the modulation effect of acupuncture intervention. Based on the method of ICA, the result revealed the existence of statistically independent LFPN, RFPN, aDMN, pDMN, SMN, VN, and SN during resting state, among of which, bilateral FPN, DMN, and SN belonged to cognitive network while SMN and VN belonged to the category of sensory perception. The main brain regions of these brain networks were consistent with previous reports.

Previous studies mostly focused on the changes of FC of RSNs in stroke patients.^[21–23]^ SMN was one of the earliest studies networks. Van and colleagues have approved that the increased interhemispheric FC of the SMN has been associated with motor recovery.^[35]^ DMN mainly participates in the function of cognitive control and emotion management.^[14]^ The dysfunction of DMN FC may underlie the cognitive impairment and abnormal emotion, such as poststroke dementia and depression.^[36]^ Further finding from another research showed that the intranetwork FC of DMN (aDMN and pDMN) presented complex changes in stroke patients, increased connectivity were found in pDMN while both increased and decreased connectivity in aDMN.^[37]^ A new research demonstrated that subcortical stroke may induce connectivity changes (intranetwork and internetwork) in multiple functional networks including SMN, VN, AN, dorsal attention network (DAN), DMN, and FPN.^[37]^ However, a relatively small number of studies concentrated on the interaction of RSNs. GCA is a special method to explore causal interactions between brain regions or networks.^[38]^ Some rehabilitation schemes can lead to decreased activity of contralesional M1, increased asymmetry of M1 activity to the ipsilesional side, decreased perilesional activity, and decreased SMA activity.^[39]^ Disruption of EC between subcortical and cerebral cortex was also found in stroke patients with motor deficit.^[40]^ In this study, we found that stroke patients with hemiplegia displayed more complex causal connection between multiple brain networks in the resting state compared with healthy subjects. Among these causal flows, SMN, DMN, and FPN were the causal hubs, SMN and DMN outputted most information to other networks while FPN inputted most information from other networks. SMN respectively sent the information flow to LFPN, aDMN, RFPN, SN, and other cognitive networks, which reflected that stroke patients with hemiplegia may transmit information by thinking and decision-making of DMN and FPN, and SN focusing on the significant information.

In this study, we also observed that acupuncture Yanglingquan could regulate multiple brain networks of stroke patients, although the modulation effects of acupuncture on DMN and SMN has been reported in our previous studies.^[41,42]^ The effect of acupuncture is often considered as the complex response pattern through the coordination between multiple brain networks. A previous study showed SMN was the most critical and core network responsible for the task launching and executing.^[43]^ The primary motor cortex and premotor cortex located in precentral gyrus were the most important brain area in motor initiation and motor control, however the primary somatosensory cortex belonging to the postcentral gyrus played a critical role in sensation input.^[37,44]^ As the important brain region of conscious movement intentions, inferior parietal lobule anatomically connected to the premotor cortex has been considered to be the area of sensory information processing and sensorimotor information integration.^[45]^ DMN is the most basic network in human, with the characteristics of positive activation under resting state while negative activation under task stimulation.^[14]^ Raichle and colleagues mentioned that DMN may play an important role in maintaining the awakening of human awareness and emotional memory.^[14]^ The medial temporal lobe and posterior cingulate cortex (PCC) were the main node of DMN, which was further divided into 2 subnetworks (aDMN and pDMN) coparticipating in information integration and modulation.^[46]^ FPN is halfway between DMN and DAN, and mainly involved in cognition controlling function such as memory, attention, and visual processing by the 2 mirroring networks (LFPN and RFPN).^[47,48]^ Superior parietal lobule is considered to be the core of the network nodes.^[48]^ VN is involved in visual attention.^[49]^ SN mainly carries on the processing to the dominant information, with antagonism to DMN.^[50]^ The changes of these networks have been found during the recovery of stroke patients.

Compared with preacupuncture, acupuncture reversed the direction between LFPN and aDMN, pDMN, SN, between pDMN and LFPN, RFPN, and SN. Then LFPN converted into the network of outputting most information from the network inputting most information before acupuncture intervention, while pDMN became the network inputting most information. It is interesting to note that LFPN outputted information to aDMN, then aDMN outputted the information to SMN, which formed the part of the information transmission loop after acupuncture. And the causal relationship existed significant differences between LFPN and aDMN (*P* <.05). Our analysis indicated that DMN, a key network, plays a critical and causal role in FPN and SMN, which may be the potential mechanism of the modulatory effect of acupuncture mainly involved the function of cognition, motor, and perception for stroke patients with hemiplegia. These results were consistent with previous studies that detected FC, interaction effect, and EC of motor-related regions in stroke patients.^[27,41,42]^ For example, the effect of acupuncture GB34 on the inter-regional interactions between the anterior cingulate cortex and PCC belonging to DMN has been reported in our previous study.^[41]^ Other scholars also confirmed the core role of DMN in the RSNs.^[51]^ Based on the multivariate GCA, the bidirectional EC between the cerebellum and primary sensorimotor cortex in stroke patients could be enhanced by acupuncture at GB34.^[27]^ Taken together, the results indicated that acupuncture probably integrated the EC internetwork of stroke patients with hemiplegia by mainly affecting the RSNs related to cognitive, motor, and perceptional function.

We only conducted the preliminary study on the potential mechanism of acupuncture in promoting RSNs reconfiguration of apoplectic hemiplegia. There are several limitations in this study. First, we did not establish sham acupuncture group because most Chinese people accepted acupuncture treatment and be relative sensitive to the feeling of acupuncture, but other acupoint such as Zusanli (ST36) could be selected as a positive control in the future study. Second, this study focused only on the resting-state brain networks without structural networks, however, the effect of acupuncture is often considered as the complex response pattern that relates to multisystem, multimodal imaging technology will be the main trend of research field in brain plasticity of stroke patient with hemiplegia. Another limitation was that we did not investigate the links between EC changes and behavioral improvements due to preliminarily explore the instant effect of acupuncture. Also, the method of GC has some shortcomings for the inherent uncertainty regarding the hemodynamic response function and its variation across different brain regions.^[52]^ Further studies should be improved in research design and analysis method, as well as combining imaging data with behavioral data may help to validate our findings and to investigate their clinical significance in hemiplegia rehabilitation.

## 5 Conclusion

In conclusion, we found that acupuncture at GB34 can regulate multiple brain networks of stroke patients with hemiplegia, and probably integrate the EC internetwork by transferring information between advanced cognitive network and SMN by DMN as the relay station. Our results demonstrated that acupuncture can induce reorganization of RSNs, which may help to understand the mechanism of acupuncture treatment for promoting hemiplegia rehabilitation. The further studies with larger sample size and multimodal analysis should be considered.

## Supplementary Material

Supplemental Digital Content
