# Supplementary material for: Altered effective connectivity of resting state networks by acupuncture stimulation in stroke patients with left hemiplegia: A multivariate granger analysis
Source: Medicine (Baltimore). 2017 Nov 27;96(47):e8897. doi: 10.1097/MD.0000000000008897 (PMC5709020; doi:10.1097/MD.0000000000008897)
Supplement: Supplemental Digital Content [file medi-96-e8897-s001.doc]

**Title:**

**Altered effective connectivity of resting state networks by acupuncture stimulation in stroke patients with left hemiplegia: A multivariate Granger analysis**

**Authors:**

Cai-Hong Fu, PhD; Kuang-Shi Li, MD, Yan-Zhe Ning, MD, Zhong-Jian Tan, MD, Yong Zhang PhD, Hong-Wei Liu PhD, Xiao Hana, MD, Yi-Huai Zou, PhD

**Affiliation/address:**

From Department of Neurology and Stroke Center, Dongzhimen Hospital, the First Affiliated Hospital of Beijing University of Chinese Medicine, Beijing, China (FC, NY; ZY, LH, HX, ZY); Shunyi Hospital Affiliated to Beijing Hospital of Traditional Chinese Medicine, Beijing, China (FC, LH); Department of Emergency, Beijing GuLou Hospital of Traditional Chinese Medicine, Beijing, China (LK); Department of Radiology, Dongzhimen Hospital, the First Affiliated Hospital of Beijing University of Chinese Medicine, Beijing, China (TZ).

**Corresponding author:** Yihuai Zou, Department of Neurology and Stroke Center, Dongzhimen Hospital, the First Affiliated Hospital of Beijing University of Chinese Medicine, Beijing 100700, China

E-mail: [zouyihuai2004@163.com](mailto:zouyihuai2004@163.com)

Tel.: 86-13681372319

**Supplementary Table. Spatial positional distributions of resting-state networks**

| **network** | **Brain regions** | **Side** | **Brodmann areas** | **Talairach** | | | ***t*-value** | **Voxels** |
| --- | --- | --- | --- | --- | --- | --- | --- | --- |
|  |  |  |  | ***X*** | ***Y*** | ***Z*** |  |  |
| SMN | Precentral Gyrus | L | 4,6 | -24 | -23 | 59 | 9.3753 | 477 |
|  | Precentral Gyrus | R | 4,6 | 30 | -29 | 57 | 7.8641 | 355 |
|  | Paracentral Lobule | L | 3,4,5,6,31 | -12 | -29 | 57 | 7.5307 | 143 |
|  | Paracentral Lobule | R | 3,4,5,6,7,31 | 6 | -29 | 57 | 8.3533 | 172 |
|  | Postcentral Gyrus | L | 1,2,3,4,5,7,40 | -24 | -29 | 54 | 9.1245 | 388 |
|  | Postcentral Gyrus | R | 1,2,3,4,5,40 | 27 | -29 | 59 | 8.1312 | 311 |
|  | Superior Frontal Gyrus | L | 6 | -3 | 6 | 55 | 7.341 | 108 |
|  | Superior Frontal Gyrus | R | 6 | 3 | 8 | 52 | 6.5914 | 79 |
|  | Middle Frontal Gyrus | L | 6 | -33 | -3 | 50 | 7.825 | 272 |
|  | Middle Frontal Gyrus | R | 6 | 18 | -9 | 58 | 7.1141 | 239 |
|  | Cingulate Gyrus | L | 24,31,32 | -3 | -4 | 44 | 6.7057 | 98 |
|  | Cingulate Gyrus | R | 24,31,32 | 3 | -1 | 44 | 6.0406 | 90 |
| VN | Middle Occipital Gyrus | L | 18,19 | -27 | -81 | 21 | 7.0904 | 107 |
|  | Middle Occipital Gyrus | R | 18,19,37 | 24 | -83 | 21 | 6.5646 | 109 |
|  | Cuneus | L | 7,17,18,19,23,30 | -18 | -83 | 32 | 9.7247 | 575 |
|  | Cuneus | R | 7,17,18,19,23,30 | 6 | -80 | 29 | 9.6234 | 606 |
|  | Fusiform Gyrus | L | 19 37 | -21 | -67 | -7 | 5.3105 | 51 |
|  | Fusiform Gyrus | R | 19 37 | 24 | -53 | -7 | 5.7667 | 91 |
|  | Lingual Gyrus | L | 17 18 19 | -12 | -64 | 3 | 6.6135 | 309 |
|  | Lingual Gyrus | R | 17 18 19 30 | 18 | -64 | 3 | 6.6008 | 303 |
| Continued | | | | | | | | |
| LFPN | Precentral Gyrus | L | 4,6,9,44,43 | -45 | 1 | 28 | 7.4726 | 315 |
|  | Superior Parietal Lobule | L | 7 | -33 | -59 | 44 | 8.9285 | 163 |
|  | Superior Parietal Lobule | R | 7 | 30 | -65 | 45 | 4.6845 | 25 |
|  | Inferior Parietal Lobule | L | 2,7,39,40 | -33 | -56 | 44 | 10.2652 | 671 |
|  | Inferior Parietal Lobule | R | 7,39,40 | 33 | -59 | 39 | 5.2633 | 133 |
|  | Middle Frontal Gyrus | L | 6,9,10,11,46,47 | -50 | 19 | 27 | 8.3426 | 203 |
|  | Middle Frontal Gyrus | R | 10,11,46,47 | 42 | 33 | 15 | 4.8534 | 139 |
|  | Inferior Frontal Gyrus | L | 9,10,44,45,46,47 | -48 | 10 | 19 | 10.7731 | 251 |
|  | Superior Temporal Gyrus | L | 22,39,42 | -36 | -54 | 28 | 6.3661 | 63 |
|  | Middle Temporal Gyrus | L | 39 | -33 | -60 | 8 | 7.0116 | 20 |
|  | Cingulate Gyrus | L | 32 | -3 | 25 | 35 | 5.6647 | 66 |
|  | Insula | L | 13 | -39 | 7 | 16 | 6.2046 | 31 |
|  | Precuneus | L | 7,19,39 | -27 | -68 | 39 | 9.9509 | 164 |
|  | Precuneus | R | 7,19,39 | 30 | -65 | 34 | 5.1028 | 48 |
| RFPN | Superior Frontal Gyrus | L | 8,9,10 | -15 | 45 | 23 | 5.6106 | 332 |
|  | Superior Frontal Gyrus | R | 8,9,10,11 | 15 | 64 | 5 | 5.0037 | 168 |
|  | Middle Frontal Gyrus | L | 6,8,9,10 | -30 | 28 | 32 | 5.2112 | 185 |
|  | Medial Frontal Gyrus | L | 6,8,9,10,11 | -3 | 50 | 11 | 5.8559 | 248 |
|  | Medial Frontal Gyrus | R | 6,8,9,10,11,25 | 3 | 56 | 11 | 5.6754 | 233 |
|  | Inferior Parietal Lobule | L | 7,39,40 | -42 | -65 | 39 | 7.2382 | 138 |
| Continued | | | | | | | | |
| RFPN | Inferior Parietal Lobule | R | 7,39,40 | 48 | -59 | 39 | 6.0337 | 121 |
|  | Superior Temporal Gyrus | L | 13,21,22,29,38 39 41 | -53 | -54 | 25 | 10.3327 | 323 |
|  | Superior Temporal Gyrus | R | 13,22,39,41,42 | 53 | -54 | 25 | 8.0651 | 303 |
|  | Middle Temporal Gyrus | L | 19,21,37,38,39 | -45 | -66 | 28 | 11.5784 | 666 |
|  | Middle Temporal Gyrus | R | 19,21,22,37,39 | 48 | -60 | 28 | 7.1933 | 196 |
|  | Anterior Cingulate | L | 24,32,42 | -3 | 47 | 9 | 3.7474 | 41 |
|  | Anterior Cingulate | R | 10,32,42 | 6 | 47 | 6 | 5.2478 | 25 |
|  | Cingulate Gyrus | L | 23,24 | -3 | -48 | 27 | 11.8944 | 198 |
|  | Cingulate Gyrus | R | 23,24,31 | 3 | -45 | 27 | 11.17 | 209 |
|  | Posterior Cingulate | L | 23,29,30,31 | -3 | -51 | 22 | 12.6884 | 226 |
|  | Posterior Cingulate | R | 23,29,30,31 | 3 | -51 | 22 | 11.0067 | 213 |
|  | Angular Gyrus | L | 39 | -42 | -68 | 31 | 9.7512 | 126 |
|  | Angular Gyrus | R | 39 | 48 | -59 | 36 | 7.2225 | 73 |
|  | Supramarginal Gyrus | L | 39,40 | -53 | -51 | 27 | 8.5929 | 134 |
|  | Supramarginal Gyrus | R | 40 | 48 | -57 | 30 | 6.5969 | 107 |
|  | Precuneus | L | 7,19,31,39 | -3 | -63 | 31 | 11.7995 | 587 |
|  | Precuneus | R | 7,19,23,31 | 3 | -59 | 39 | 11.8242 | 409 |
| aDMN | Superior Frontal Gyrus | L | 8,9,10 | -15 | 34 | 43 | 8.4859 | 95 |
|  | Superior Frontal Gyrus | R | 6,8,9,10 | 6 | 20 | 57 | 6.9091 | 101 |
|  | Medial Frontal Gyrus | L | 8,9,10,32 | -3 | 55 | 3 | 9.313 | 139 |
| Continued | | | | | | | | |
| aDMN | Medial Frontal Gyrus | R | 8,9,10 | 3 | 48 | 20 | 7.7106 | 83 |
|  | Anterior Cingulate | L | 24,32,42 | -3 | 41 | 3 | 7.1814 | 85 |
|  | Anterior Cingulate | R | 24,32,42 | 3 | 38 | 9 | 6.9717 | 30 |
|  | Cingulate Gyrus | L | 24,32 | -3 | 19 | 32 | 6.9196 | 27 |
|  | Cingulate Gyrus | R | 32 | 3 | 22 | 32 | 6.2497 | 13 |
| pDMN | Superior Frontal Gyrus | R | 6,8,9,10,11 | 21 | 20 | 43 | 6.5477 | 379 |
|  | Middle Frontal Gyrus | R | 6,8,9,10,11,46,47 | 45 | 25 | 35 | 7.9115 | 1018 |
|  | Medial Frontal Gyrus | L | 6,9 | -3 | 42 | 31 | 4.4327 | 29 |
|  | Superior Temporal Gyrus | R | 13,22,39,41 | 53 | -57 | 28 | 9.1536 | 214 |
|  | Middle Temporal Gyrus | R | 19,20,21,22,39 | 50 | -60 | 28 | 7.9976 | 256 |
|  | Superior Parietal Lobule | R | 7 | 36 | -62 | 45 | 9.1056 | 126 |
|  | Inferior Parietal Lobule | L | 7,39,40 | -45 | -59 | 39 | 5.3865 | 141 |
|  | Inferior Parietal Lobule | R | 7,39,40 | 50 | -48 | 38 | 10.7813 | 684 |
|  | Cingulate Gyrus | L | 23,31,32 | -3 | -30 | 32 | 5.3899 | 29 |
|  | Cingulate Gyrus | R | 23,24,31,32 | 3 | -33 | 35 | 5.2428 | 192 |
|  | Precuneus | R | 7,19,31,39 | 33 | -71 | 42 | 8.843 | 383 |
| SN | Precentral Gyrus | L | 6,13,42,44 | -56 | 9 | 8 | 9.0312 | 56 |
|  | Precentral Gyrus | R | 4,6,13,43,44 | 50 | -8 | 6 | 8.5767 | 68 |
|  | Postcentral Gyrus | R | 2,40,43 | 59 | -22 | 20 | 12.8732 | 96 |
|  | Inferior Frontal Gyrus | L | 13,44,45,46,47 | -33 | 23 | -9 | 10.2672 | 295 |
| Continued | | | | | | | | |
| SN | Inferior Frontal Gyrus | R | 13,44,45,47 | 53 | 14 | -3 | 11.2265 | 244 |
|  | Superior Temporal Gyrus | L | 13,21,22,29,38 39 41 42 | -59 | -26 | 10 | 12.5252 | 729 |
|  | Superior Temporal Gyrus | R | 13,21,22,29,38 39 41 42 | 53 | -46 | 11 | 12.3714 | 798 |
|  | Middle Temporal Gyrus | L | 19,21,22,37,39 | -59 | -3 | -2 | 9.6531 | 219 |
|  | Middle Temporal Gyrus | R | 19,21,22,37,39 | 50 | -43 | 8 | 12.3703 | 532 |
|  | Cingulate Gyrus | L | 24,31,32 | -6 | -10 | 39 | 7.6592 | 60 |
|  | Supramarginal Gyrus | R | 40 | 56 | -39 | 32 | 8.7339 | 81 |
|  | Insula | L | 13,22,40 | -42 | 3 | 0 | 11.2432 | 285 |
|  | Insula | R | 13,22,29,40,47 | 36 | 0 | -3 | 12.0749 | 311 |
